# Predictors of Integration Among International Medical Students in Hungary

**DOI:** 10.1007/s40670-026-02767-8

**Published:** 2026-05-26

**Authors:** Regina Molnár, Afriza Umami, Andrea Szabó, Mária Kucsera, Zsuzsanna Máté, Nóra Főző, Edit Paulik

**Affiliations:** 1 Department of Public Health, Albert Szent-Györgyi Medical School, University of Szeged, Szeged, Hungary; 2 Stikes Muhammadiyah Bojonegoro, Bojonegoro, Indonesia; 3 Albert Szent-Györgyi Medical School, University of Szeged, Szeged, Hungary

**Keywords:** Acculturation, Health Behaviours, Integration, International Medical Students, Well-being

## Abstract

**Background:**

International medical students are exposed to complex cultural and academic environments that require continuous adaptation to both their host society and their culture of origin. This study examines acculturation among international medical students in Hungary using Berry’s Acculturation Theory, which classifies individuals into four categories: Integration, Separation, Assimilation, and Marginalization.

**Methods:**

A cross-sectional study was conducted among 326 international medical students using a modified Stephenson Multigroup Acculturation Scale and self-reported measures of health behaviours, perceived stress, and mental well-being. Most participants were categorized as either Integration (46.0%) or Separation (50.9%), while only a small portion of students fell into Assimilation (0.3%) or Marginalization (2.8%) category. Consequently, the analysis focused on comparing the Integration and Separation groups.

**Results:**

Significant differences were observed in demographic and behavioural characteristics between these groups. Males (adjusted odds ratio [aOR] = 1.88, *p* = 0.013), preclinical students (aOR = 1.78, *p* = 0.048), non-drinking students (aOR = 2.57, *p* = 0.001), and those with lower perceived stress (aOR = 2.15, *p* = 0.035), integrated stronger. Meanwhile, non-smokers (aOR = 0.52, *p* = 0.040) and those who abstain from psychologist visits (aOR = 0.36, *p* = 0.008) integrated less well.

**Conclusion:**

The present study identifies demographic and health-related factors associated with integration among international medical students. These findings may contribute to a better understanding of acculturation patterns and inform strategies to support students’ social adjustment and well-being.

**Supplementary Information:**

The online version contains supplementary material available at https://doi.org/10.1007/s40670-026-02767-8.

## Introduction

### Acculturation Strategies and Influencing Factors

Acculturation refers to the psychological and cultural adjustments that individuals undergo when exposed to a new culture and the strategies they may adopt to navigate the complexities of cultural integration [1, 2].

John W. Berry’s model (2004) categorizes individuals’ responses to their new cultural environment into two distinct orientations. These categories are based on the extent to which individuals maintain their culture of origin and engage with the host society. Different combinations of high or low involvement give rise to four acculturation strategies: individuals may participate in both cultures (integration), maintain the culture of origin while limiting engagement with the host society (separation), adopt the host culture while relinquishing the culture of origin (assimilation), or show limited involvement in both cultural contexts (marginalization) [3–6].

Acculturation strategies are influenced by a variety of predictors that encompass individual characteristics, social factors, and contextual elements [7, 8], which vary significantly across host countries [9]. Berry’s model of acculturation provides a framework for understanding how these predictors shape the choices individuals make regarding their cultural integration [3, 10].

Berry argued that integration is typically the most adaptive strategy, leading to better psychological outcomes and reduced acculturative stress compared to other strategies [2, 11]. This perspective is supported by various studies indicating that individuals who integrate their cultural identities experience less stress [12, 13] and greater life satisfaction [14–16]. Such integration has been associated with positive psychological outcomes, including lower stress and better adaptation [10, 17].

A previous study found that better integration into ethnic groups correlated with improved general health, while stronger integration into the host country was linked to better mental health outcomes [18]. Conversely, separation and marginalization are often linked to higher stress and social isolation, while assimilation may limit individuals’ connections to their cultural roots [5, 19, 20].

Studies on international students indicate that the acculturation process is influenced by factors such as social support [21, 22], personality traits, language proficiency [23], perceived discrimination, academic outcomes [24, 25], and prior exposure to the host culture [17].

### Acculturation Among International Students

The number of international students in Hungary has increased significantly over the past decade. In the 2017/18 academic year, studying medicine was the most popular study program among international students: about 30% of the total number was enrolled in medicine and dentistry [26, 27].

The complexity of acculturation among international medical students in Hungary primarily arises from the linguistic challenges they face. Many programs are offered in English or German to accommodate non-Hungarian speakers [28]. In contrast, in clinical training patients often don’t speak English and daily interactions outside the classroom frequently require speaking in Hungarian [27, 28]. Hungarian universities also provide structured Hungarian language courses to aid international students. These programs often focus on medical Hungarian and patient–doctor communication to bridge the gap between language learning and practical applications in healthcare [29–31].

International students also operate within a multilingual social environment, often interacting with peers from diverse cultural and linguistic backgrounds. This dynamic compels them to communicate in English, while also maintaining ties to their native languages.

However, many studies have addressed how health behaviours (e.g., stress, smoking, alcohol use) and demographic factors interact with acculturation categories in this cohort, predominantly in countries with large international student populations such as the United States [7, 32]. This leaves a gap in understanding acculturation dynamics in smaller European countries such as Hungary, where cultural and academic contexts may differ significantly. Medical education is academically demanding and requires a prolonged period of residence, with international medical students typically spending a minimum of six years in the host country. During this extended and intensive training period, students must not only meet high academic expectations but also navigate unfamiliar cultural and social environments.

Consequently, the ability to adopt effective acculturation strategies—particularly integration—may play a crucial role in students’ academic performance, psychological well-being, and long-term adaptation, whereas difficulties in acculturation may increase vulnerability to stress and social isolation.

This study seeks to fill these gaps by applying Berry’s model to a diverse group of international medical students in Hungary. It explores not only the distribution of acculturation strategies but also the relationships between demographic variables (e.g., sex, academic year) and health behaviours associated to integration and separation outcomes. Our study aims to contribute to the growing body of knowledge on acculturation patterns in international educational contexts and highlight the potential health implications associated with different acculturation categories.

## Methods

### Study Design and Participants

This study was part of a larger research project conducted among international medical students at the University of Szeged, Hungary and followed a cross-sectional design. Data were collected between April and October 2021. A total of 326 international medical students completed the questionnaire.

### Variables and Measurement

#### Dependent Variable: Acculturation Categories

Acculturation was measured using the modified Stephenson Multigroup Acculturation Scale (SMAS). This instrument evaluates two dimensions: Ethnic Society Immersion (ESI) reflects the retention of values and practices from the home culture and Dominant Society Immersion (DSI) measures the adoption of values and practices from the host culture [33].

The questionnaire was administered in English. All participants were enrolled in English- or German-language medical programs, where sufficient English proficiency is required. However, variability in language proficiency levels among participants was not formally assessed.

The modified 28-item SMAS questionnaire was adapted to exclude items related to English language ability, as English proficiency was a study inclusion criterion. The exclusion of language-related items does not alter the conceptual structure of the SMAS, but may influence the distribution of scores, particularly for the DSI dimension. Therefore, in the present study, DSI primarily reflects social and behavioural engagement with the host society rather than linguistic integration.

The ESI scale contained 16 items, and the DSI scale included 12 items. Responses were recorded on a Likert scale (1 = false to 4 = true), with higher scores indicating greater immersion in either dimension. The items of the questionnaire are available in the Supplementary Information (Additional file 1) and from Internal consistency reliability was assessed for both subscales, yielding satisfactory Cronbach’s alpha coefficients (α = 0.82 for ESI and α = 0.88 for DSI), supporting the internal reliability of the modified instrument in the present sample (see Additional file 2).

Participants were categorized into Berry’s four acculturation groups based on sample-specific mean ESI and DSI scores. Thresholds for categorizing “high” and “low” ESI and DSI were derived from the mean values observed in the present study sample (ESI ≥ 3.19 and DSI ≥ 2.05). While this approach is consistent with mean-based classification strategies used in previous studies, the exact cut-off values in this study are sample-specific. Although this approach is consistent with mean-based classification methods used in previous studies, the exact cut-off values are specific to this sample (Asif, 2018): Integration: High ESI (≥ 3.19) and High DSI (≥ 2.05). Separation: High ESI (≥ 3.19) and Low DSI (< 2.05). Assimilation: Low ESI (< 3.19) and High DSI (≥ 2.05). Marginalization: Low ESI (< 3.19) and Low DSI (< 2.05).

In this study, Dominant Society Immersion (DSI) primarily reflects behavioural and social engagement with the host society, including social interactions and cultural familiarity, rather than linguistic integration.

#### Independent Variables

Demographic characteristics: age was measured as a continuous variable (years); sex was categorized as “male” or “female”; relationship status was dichotomized as “not in relationship” (single, divorced, living separately) and “in relationship” (married, common-law marriage, living together, having a partner but not living together). Years of study were categorized as “preclinical years” (1st, 2nd ) and “clinical years” (3rd, 4th, 5th, 6th ). The economic condition of the students’ family was evaluated by a 5-point Likert scale, and it was dichotomized as “low income” (very bad, bad, average) and “high income” (good, very good). Ethnic minority status was assessed by asking students whether they considered themselves members of an ethnic minority group, with response options of “Yes” or “No”. Religion was categorized into four groups: Christian, Islam, other religions, and neither. For other religions, Jewish, Buddhist, Hindu, etc. were combined into one category due to the small number of participants.

Health behaviours: Smoking: the response options were “Yes, occasionally”; “Yes, regularly”; and “No”. In the analysis both occasional and regular smokers were considered as smokers. Alcohol consumption: the response options were “Yes, occasionally”; “Yes, regularly”; and “No”. In the analysis both occasional and regular alcohol consumers were considered as alcohol drinkers.

Health status: Self-rated Health (SRH) was reported by using a five-point Likert scale (1 = very bad, 5 = very good). The respondents had to answer the question regarding general health, “How do you evaluate your general health status?” For the purposes of data analyses, SRH was categorized as good (scores 4 and 5) and poor (scores 1 to 3). Self-rated mental health (SRMH) was reported by using a five-point Likert scale (1 = very bad, 5 = very good). The respondents had to answer the question regarding mental health, “How do you evaluate your mental health status?” For the purposes of data analyses, SRMH was categorized as good (scores 4 and 5) and poor (scores 1 to 3).

Perceived Stress: The 10-item Perceived Stress Scale (PSS) was originally developed by Cohen et al. (1983). The questions in the PSS ask about feelings and thoughts during the last month. It is a measure of the degree to which situations in one’s life are appraised as stressful. Items were designed to tap how unpredictable, uncontrollable, and overloaded respondents find their lives. The scale also includes a number of direct queries about current levels of experienced stress from “never” to “very often”. The PSS is not a diagnostic instrument and individual scores on the PSS can range from 0 to 40 with higher scores indicating higher perceived stress. Scores between 0 and 13 were considered low stress, between 14 and 26 moderate stress and between 27 and 40 high perceived stress [34]. In the current study, for the purposes of data analysis, PSS was categorized into two groups: lower stress (a low stress score of 0–13) and higher stress (moderate and high stress score of 14–40).

Visit to Psychologist: students answered whether they visited a psychologist during their studies, and the response options were “Yes” or “No”.

#### Statistical Analysis

Descriptive statistics, including frequencies, percentages, means, and standard deviations, were calculated for sample characteristics. Comparison of demographic and health characteristics between the Separation and Integration groups was conducted using the Chi-square test for categorical variables and the Mann-Whitney U test for continuous variables.

Univariable logistic regression assessed associations between dependent variables and covariates. Multivariable logistic regression was used to evaluate the independent effects of acculturation (ESI and DSI scores) on outcomes while adjusting for covariates. Cross-classifications of ESI and DSI scores were used to validate Berry’s acculturation categories. The Hosmer–Lemeshow test assessed model fit. Odds ratios (OR) and 95% confidence intervals (CI) quantified associations. Statistical significance was set at *p* < 0.05.

Variables included in the multivariable logistic regression model were selected based on theoretical relevance and prior literature, rather than solely on statistical significance in univariable analyses, to ensure appropriate adjustment for potential confounding. Multicollinearity among independent variables was assessed using variance inflation factors (VIF), with all values below 2 (range: 1.04–1.47), indicating no significant collinearity among predictors (Additional file 3).

Because the number of participants classified as Assimilation and Marginalization was very small, these groups were excluded from further analysis. Therefore, the multivariable analyses focused on comparing the Integration and Separation groups.

All analyses were performed using IBM SPSS Statistics for Windows, version 28 (SPSS Inc., Chicago, IL, USA).

## Results

### Participants’ Characteristics

Table 1 provides an overview of the participants’ demographics, health status, and behaviours. The study primarily involved young adults, with a slightly higher representation of females than males. More participants were in preclinical years of their studies and reported a higher economic status. A significant portion of the group was not in a relationship, and the majority did not identify as part of an ethnic minority. Regarding religion, Christianity and Islam were the most common, followed by other religions.

**Table 1:** Characteristics of participants (*N* = 326)

Characteristics	*n*	(%)
Age (years) Mean (SD)	22.8	(2.8)
Sex		
Male	151	46.3
Female	175	53.7
Year of study		
Preclinical	224	68.7
Clinical	102	31.3
Economic status		
Lower	86	26.4
Higher	240	73.6
Relationships status		
not in relationship	244	74.8
in relationship	82	25.2
Ethnic minority		
No	278	85.3
Yes	48	14.7
Religion		
Christian	136	41.7
Islam	98	30.1
Other religions	71	21.8
Neither	21	6.4
Smoking		
No	248	76.1
Yes	78	23.9
Alcohol		
No	126	38.7
Yes	200	61.3
General health		
Good	220	67.5
Poor	106	32.5
Mental health		
Good	164	50.3
Poor	162	49.7
PSS		
Lower	269	82.5
Higher	57	17.5
Visit psychologist		
No	280	85.9
Yes	46	14.1

*PSS* Perceived Stress Scale, *SD* Standard Deviation

In terms of health, many participants rated their general health positively, though mental health outcomes were mixed, with a notable proportion experiencing poor mental health. Non-smoking was prevalent among participants, although alcohol consumption was relatively common. Most of them reported low stress levels, and only a small number had sought psychological support.

### Classification of SMAS in Relation to Berry’s Theory

#### Acculturation Categories

As shown in Table 2, the majority of participants (96.9%) demonstrated high ESI (scores ≥ 3.19), indicating a strong connection to their ethnic community, while only 3.1% scored low. In contrast, DSI scores were more evenly distributed, with 53.7% showing low immersion in the dominant society (scores < 2.05) and 46.3% exhibiting high immersion.

**Table 2:** Dichotomy of ESI and DSI (*N* = 326)

Characteristics	Total (326)	
	*n*	%
ESI		
Low (score < 3.19)	10	3.1
High (score ≥ 3.19)	316	96.9
DSI		
Low (score < 2.05)	175	53.7
High (score ≥ 2.05)	151	46.3

*ESI* Ethnic Society Immersion, *DSI* Dominant Society Immersion

Based on Berry’s Acculturation Theory, participants were categorized into four acculturation groups (Table 3). The largest group (50.9%) was classified as Separation, characterized by high ESI and low DSI, reflecting a preference for ethnic community engagement while distancing from the dominant society. The second largest group (46.0%) fell into Integration, where participants displayed high scores in both ESI and DSI, indicating active participation in both ethnic and dominant communities. A smaller proportion (2.8%) was categorized as Marginalization, with low scores in both ESI and DSI, signifying limited connection to both societies. Lastly, only 0.3% were classified as Assimilation, demonstrating high DSI and low ESI, which indicates a preference for the dominant society over their ethnic identity.

**Table 3:** Two-Dimensional Acculturation Models

Ethnic society immersion	Dominant society immersion	
	Low	High
High	Separation 166 (50.9%)	Integration 150 (46.0%)
Low	Marginalization 9 (2.8%)	Assimilation 1 (0.3%)

The comparison of demographic and health characteristics between Separation and Integration groups highlighted several key differences (Table 4). Sex distribution showed a significantly higher proportion of males in the Integration group (52.7%) compared to the Separation group (41.0%, *p* = 0.037). Regarding year of study, a greater percentage of preclinical students were in Integration group (75.3%) compared to Separation (64.5%, *p* = 0.036). Religion also differed significantly, with more Christians in the Integration group (52.0%) than in Separation group (34.3%, *p* = 0.017). For PSS, individuals in Integration group were more likely to report lower stress levels (88.0%) compared to those in Separation (77.1%, *p* = 0.011). Lastly, while not statistically significant, fewer individuals in the Integration group visited a psychologist (17.3%) compared to the Separation group (10.8%, *p* = 0.096).

**Table 4:** Comparison of Demographic and Health Characteristics Between Separation and Integration Groups (Chi-square and Mann-Whitney U)

Variables	Integration (*N* = 150) *n* (%)	Separation (*N* = 166) *n* (%)	*p*-value
Age (years) Mean±SD	23.01±3.0	22.72±2.6	0.549
Sex			
Male	79 (52.7)	68 (41.0)	**0.037**
Female	71 (47.3)	98 (59.0)	
Year of study			
Preclinical	113 (75.3)	107 (64.5)	**0.036**
Clinical	37 (24.7)	59 (35.5)	
Economic status			
Lower	40 (26.7)	41(24.7)	0.689
Higher	110 (73.3)	125 (75.3)	
Relationships status			
Not in relationship	108 (72.0)	126 (75.9)	0.429
In relationship	42 (28.0)	40 (24.1)	
Ethnic minority			
No	130 (86.7)	142 (85.5)	0.773
Yes	20 (13.3)	24 (14.5)	
Religion			
Christian	78 (52.0)	57 (34.3)	**0.017**
Islam	38 (25.3)	55 (33.1)	
Other religions	26 (17.3)	41 (24.7)	
Neither	8 (5.3%)	13 (7.8)	
Smoking			
No	110 (73.3)	133 (80.1)	0.153
Yes	40 (26.7)	33 (19.9)	
Alcohol			
No	64 (42.7)	57 (34.3)	0.128
Yes	86 (57.3)	109 (65.7)	
General health			
Good	108 (72.0)	108 (65.1)	0.185
Poor	42 (28.0)	58 (34.9%)	
Mental health			
Good	85 (56.)	77 (46.4)	0.068
Poor	65 (43.3)	89 (53.6)	
PSS			
Lower	132 (88.0)	128 (77.1)	**0.011**
Higher	18 (12.0)	38 (22.9)	
Visit psychologist			
No	124 (82.7)	148 (89.2)	0.096
Yes	26 (17.3)	18 (10.8)	

*PSS* Perceived Stress Scale, *SD* Standard Deviation

Table 5 presents the results of logistic regression analysis identifying factors associated with Integration, showing both unadjusted odds ratios (OR) and adjusted odds ratios (aOR). Several significant predictors emerged. Sex was a significant factor, males being 1.88 times more likely to integrate compared to females (aOR = 1.88, *p* = 0.013). Year of study also played a role, as preclinical students were more likely to integrate compared to those in later years (aOR = 1.78, *p* = 0.048). Smokers were more likely to adopt an integration strategy than non-smokers (aOR = 0.52, *p* = 0.040), whereas students who consumed alcohol were less likely to be integrated compared to non-drinkers (aOR = 2.57, *p* = 0.001). PSS scores indicated that lower stress levels significantly increased the odds of integration (aOR = 2.15, *p* = 0.035). Lastly, visiting a psychologist was positively associated with integration, whereas students who did not visit a psychologist had significantly lower odds of integration (aOR = 0.36, *p* = 0.008). These findings underscore the multifaceted nature of integration, influenced by demographic, behavioural, and psychological factors.

**Table 5:** Logistic Regression Analysis of Factors Associated with Integration and Separation: Unadjusted and Adjusted Odds Ratios

	OR (95% CI)	*p*- value	aOR (95% CI)	*p*- value
Age (years)	1.03 (0.95–1.12)	0.364	1.04 (0.94–1.14)	0.398
Sex				
Male	1.60 (1.02–2.50)	**0.037**	1.88 (1.14–3.10)	**0.013**
Female	1.00		1.00	
Year of study				
Preclinical	1.68 (1.03–2.74)	**0.037**	1.78 (1.00-3.17)	**0.048**
Clinical	1.00		1.00	
Economic status				
Lower	1.11(0.66–1.83)	0.689	1.32 (0.74–2.34)	0.338
Higher	1.00		1.00	
Relationships status				
Not in relationship	0.81 (0.49–1.35)	0.430	0.66 (0.37–1.18)	0.163
In relationship	1.00		1.00	
Ethnic minority				
No	1.09 (0.58–2.08)	0.773	0.61 (0.29–1.32)	0.217
Yes	1.00		1.00	
Religion				
Christian	2.22 (0.86–5.71)	0.097	2.66 (0. 89-7.96)	0.080
Islam	1.12 (0.42–2.97)	0.815	0.88 (0.29–2.66)	0.823
Other religions	1.03 (0.37–2.82)	0.953	1.03 (0. 33-3.22)	0.953
Neither	1.00		1.00	
Smoking				
No	0.68 (0.40–1.15)	0.154	0.52 (0.28–0.97)	**0.040**
Yes	1.00		1.00	
Alcohol				
No	1.42 (0.90–2.24)	0.128	2.57 (1.43–4.60)	**0.001**
Yes	1.00		1.00	
General health				
Good	1.38 (0.85–2.22)	0.186	1.03 (0.54–1.95)	0.920
Poor	1.00		1.00	
Mental health				
Good	1.51 (0.96–2.35)	0.068	1.46 (0.80–2.65)	0.215
Poor	1.00		1.00	
PSS				
Low	2.17 (1.18–4.01)	**0.013**	2.15 (1.05–4.40)	**0.035**
High	1.00		1.00	
Visit psychologist				
No	0.58 (0.30–1.10)	0.099	0.36 (0.17–0.76)	**0.008**
Yes	1.00		1.00	

*PSS* Perceived Stress Scale, *SD* Standard Deviation, *aOR* Adjusted Odds Ratios, *OR* Odds Ratios

## Discussion

Our analysis highlights significant predictors of acculturation strategies, particularly the likelihood of adopting an integration strategy. These findings are consistent with Berry’s acculturation theory, which emphasizes the role of personal and contextual factors in shaping how individuals navigate cultural integration while maintaining their ethnic identity [2].

This study identified significant sex differences in the adoption of integration strategies among international students. Specifically, male students were significantly more likely to adopt an integration strategy than female students. This finding suggests that males may encounter greater opportunities or social expectations to engage with both their ethnic community and the dominant society, facilitating integration. This pattern is supported by the broader literature. Aladegbaiye et al. (2022) highlighted the role of gender in shaping acculturation motivation among international students and noted that Berry’s model has limited capacity to fully capture gender-specific challenges faced by student migrants [35]. Similarly, Krsmanovic (2020) found that male first-year international undergraduate students in the United States were more inclined to adopt integration strategies, whereas females often faced additional barriers such as restricted social access or cultural constraints, leading to a preference for separation or marginalization strategies [36]. Hosseini-Nezhad (2023) provided further nuance by demonstrating that gender norms from students’ home cultures strongly influenced cross-cultural adaptation processes. Male students were more likely to engage with the host culture, while female students frequently experienced restrictions rooted in traditional expectations, limiting their integration opportunities [37].

The present study also revealed that students in their preclinical years were significantly more likely to adopt integration strategies compared to those in their clinical years. This finding underscores the importance of the initial years of study in fostering intercultural engagement and adaptive acculturation among international students. Previous research has shown that first-year students often demonstrate a stronger inclination toward integration as they actively navigate a new cultural environment [36]. Longitudinal evidence further suggests that acculturative stress and adjustment processes are particularly salient during the early years of study, often coinciding with heightened motivation for integration [38]. This motivation is typically strongest during the initial stages of international education, encouraging students to participate in both ethnic and host communities [35].

However, other studies present a more nuanced picture. Oduwaye et al. (2023) reported that international students frequently encounter increased challenges as they progress in their studies, with academic pressure and social isolation often hindering integration in later years [39]. Similarly, Van Mol observed that students in advanced stages of study tend to strengthen ties with their home culture while facing intensified academic demands, which may reduce engagement with local communities and weaken integration strategies [40].

We found an important relationship between behavioural factors and integration outcomes. In the present study, non-smokers were less likely to integrate, whereas non-drinkers were more likely to adopt an integration strategy. Prior research has yielded mixed findings regarding these behaviours.

In terms of the relationship between smoking and integration, a qualitative evidence suggests that smoking among university students serves important social functions beyond substance use. Cigarettes facilitate brief informal interactions and peer bonding, especially during stress, and can act as a non-verbal signal of distress that elicits social support. Because smoking is embedded in everyday social routines, smoking-related spaces may create additional opportunities for spontaneous social interaction among students [41].

Moreover, smoking is often perceived negatively within host societies, potentially limiting cross-cultural relationship formation [42]. Smoking has also been linked to reduced self-esteem, which may further constrain acculturation opportunities [43]. Nonetheless, non-smokers may experience lower integration in contexts where smoking functions as a common social practice. Krsmanovic noted that some international students experienced separation or marginalization because they did not participate in dominant social behaviours such as smoking [36]. In certain cultural settings, smoking may serve as a bonding mechanism, and abstention may inadvertently contribute to social distance [11].

These findings should be interpreted cautiously. Rather than implying that smoking facilitates integration, the observed association likely reflects broader patterns of social participation that vary across cultural and institutional contexts. Previous research has shown that international students who do not engage in visible or dominant social practices may experience greater social distance or a preference for separation [11, 36]. These dynamics are highly context-dependent, and smoking should not be regarded as a universal or normative pathway to integration.

With regard to the relationship between alcohol consumption and integration, some studies suggest that drinking is often embedded in social contexts that facilitate cross-cultural interaction and group bonding [44]. Moderate alcohol consumption may support socialization, whereas excessive drinking can lead to isolation and cultural misunderstandings [7]. Similarly, participation in social activities, including those involving alcohol, has been associated with lower acculturative stress and greater integration [22].

Other studies, however, report contrasting findings. Yans (2020) research on acculturation and well-being indicates that social drinking can enhance integration by fostering peer connections, reducing stress, and improving overall well-being [45]. Additional evidence suggests that students who engage in social drinking may find it easier to establish friendships and networks, supporting integration [44]. In contrast, smoking has been described as a less socially engaging behaviour and has been associated with increased isolation [46]. Although alcohol consumption is often associated with social contexts, recent evidence shows that it is not directly linked to social connectedness or the need to belong, but is instead influenced indirectly through cognitive expectancies and psychosocial factors [47].

Lower perceived stress was also associated with higher odds of integration among international students. Reduced stress may facilitate social participation and adaptive engagement with both host and ethnic cultures. Previous studies emphasize the importance of mental health and social support in mitigating stress and promoting integration [48, 49]. Targeted interventions addressing acculturative stressors such as homesickness and cultural differences have been shown to significantly enhance integration outcomes [50]. Consistent with this literature, Gyasi et al. highlighted the role of socio-demographic factors and culturally sensitive practices in supporting international students’ adjustment and healthcare utilization [51]. Nevertheless, the relationship between stress and integration remains complex. Persistent academic demands and social isolation in later years may undermine long-term integration despite early success [39]. Individual differences in resilience and coping strategies can also enable successful integration under high stress, indicating that stress alone does not determine acculturation outcomes [52].

The finding that students who did not visit a psychologist were less likely to integrate suggests that seeking mental health support may represent a proactive coping strategy that facilitates integration. University support systems may therefore play a critical role in helping international students manage acculturative stress and navigate cultural challenges [6]. A study among international students in China revealed that more than 80% avoided seeking healthcare services when ill, primarily due to language barriers, financial constraints, and limited awareness of health support systems [51]. The authors emphasized that institutional support, linguistic accommodations, and culturally sensitive healthcare services are crucial for improving international students’ well-being and integration [51].

Our previous study investigated the relationship between acculturation and self-rated health among international medical students in Hungary, suggesting that effective integration into both cultural contexts can enhance overall health [18]. This highlights the importance of successfully blending elements from the home culture with those of the host culture to foster better mental and physical health [12, 53]. Meta-analytic evidence indicates that social support generally buffers acculturative stress and promotes better psychological outcomes among international students [50]. However, not all forms of support are equally effective. Some studies report limited effects of social support on psychological distress, highlighting variability in student responses to available resources [22]. Cultural perceptions of mental health care may further influence service utilization, as some students prefer informal social coping strategies over professional help while still achieving successful integration [54].

### Limitations

This study has several limitations that should be considered when interpreting the findings. The cross-sectional design does not allow conclusions about cause-and-effect relationships between acculturation strategies and health-related factors. The analyses focused on identifying demographic and behavioural factors associated with acculturation; however, we did not examine whether belonging to a particular acculturation group (such as marginalization or separation) might, in turn, influence students’ health behaviours or mental well-being. Addressing these potentially bidirectional relationships would require longitudinal research.

The data were based on self-reported measures, which may be subject to reporting bias. In addition, the use of convenience sampling limits the extent to which the findings can be generalized to other student populations. Although participants came from diverse cultural backgrounds, differences between cultural subgroups were not examined in detail, which may have masked more subtle variations in acculturation experiences. Finally, the impact of academic demands and accumulated stress across different stages of medical training deserves further investigation, ideally through longitudinal study designs.

The exclusion of language-related items and the use of sample-specific cut-off values may limit comparability with other studies and could introduce potential misclassification and the language proficiency was not measured as a variable in this study. Given its well-established role in acculturation processes, the absence of this measure represents a limitation and may have influenced the observed associations.

Acculturation to the Hungarian context was assessed mainly through behavioural and social indicators, as language-related items were excluded. Therefore, the measure does not fully capture host-language acquisition, which is a central component of acculturation. Although English proficiency enables participation in academic programs, limited proficiency in the Hungarian language may hinder broader social and functional integration. Knowledge of Hungarian is likely important not only for communication with patients in clinical settings but also for everyday interactions, such as engaging with local communities, accessing services, and navigating daily life.

Further qualitative and quantitative studies are needed to analyse the role of alcohol consumption and smoking in integration. Importantly, this study does not allow causal conclusions regarding why non-smokers were less likely to integrate. Unmeasured factors such as peer group composition, cultural norms, or alternative forms of social engagement may have influenced this association.

The participation in wellness or social activities (either team-based or individual) was not assessed. Such activities may play an important role in facilitating social interaction and promoting acculturation, particularly integration into the host society. Future studies should consider including these variables to better understand their contribution to acculturation processes.

## Conclusions

This study contributes to the existing literature on acculturation by applying Berry’s acculturation framework to international medical students in Hungary and identifying demographic and health-related factors associated with integration and separation strategies. The findings indicate that integration is more prevalent among students with higher immersion in both their ethnic and the dominant cultures, a pattern consistent with Berry’s conceptualization of integration.

Observed sex differences in acculturation strategies warrant careful interpretation. Male students may experience broader social access or fewer cultural constraints in certain academic and social contexts, potentially facilitating greater interaction with both co-national and host-society peers. However, such patterns are likely shaped by culturally specific gender norms and institutional environments and should not be generalized across settings.

The association between visiting a psychologist and integration may reflect cultural differences in attitudes toward help-seeking behaviour. In some cultural contexts, psychological support is perceived as acceptable or even encouraged, while in others it may be stigmatized, potentially influencing both service utilization and broader engagement with the host society.

More broadly, the effectiveness and adaptiveness of different acculturation strategies may depend on sociopolitical and institutional contexts, including university support systems, inclusivity of the academic environment, and opportunities for intercultural interaction. Integration may be more achievable in settings that actively promote social inclusion and provide accessible support services for international students.

Given the cross-sectional nature of the study, these interpretations should be viewed as exploratory. Future research using longitudinal and mixed-methods designs is needed to better understand how gender norms, help-seeking behaviours, and institutional contexts interact with acculturation processes over time, particularly in smaller European countries.

## Electronic Supplementary Material

Below is the link to the electronic supplementary material.

Supplementary Material 1 (DOCX 26.8 KB)

## Abbreviations

aOR: Adjusted Odds Ratio
CI: Confidence Interval
DSI: Dominant Society Immersion
ESI: Ethnic Society Immersion
IMS: International Medical Students
OR: Odds Ratio
PSS: Perceived Stress Scale
SMAS: Stephenson Multigroup Acculturation Scale
SRH: Self–Rated Health
SRMH: Self–Rated Mental Health
