# Supplementary material for: Predictors of Integration Among International Medical Students in Hungary
Source: Med Sci Educ. 2026 May 26;36(4):1973–83. doi: 10.1007/s40670-026-02767-8 (PMC13582705; doi:10.1007/s40670-026-02767-8)
Supplement: Supplementary file 1 [file 40670_2026_2767_MOESM1_ESM.docx]

**SUPPLEMENTARY FILE**

Additional file 1

Table Descriptive Statistics for the Stephenson Multigroup Acculturation Scale (SMAS)

| No | Items | M±SD | False  n (%) | Partly false  n (%) | Partly true  n (%) | True  n (%) |
| --- | --- | --- | --- | --- | --- | --- |
| 1 | I speak my native language with my friends and acquaintances from my country of origin (n=326) | 3.49±0.81 | 15 (4.6) | 21 (6.4) | 79 (24.2) | 211 (64.7) |
| 2 | I have never learned to speak the language of my native country (n=326) | 1.36±0.79 | 259 (79.4) | 33 (10.1) | 19 (5.8) | 15 (4.6) |
| 3 | I eat traditional food from my native culture (n=326) | 3.33±0.86 | 20 (6.1) | 25 (7.7) | 109 (33.4) | 172 (52.8) |
| 4 | I feel comfortable speaking my native language (n=326) | 3.62±0.75 | 11 (3.4) | 20 (6.1) | 51 (15.6) | 244 (74.8) |
| 5 | I am informed about current affairs in my native country (n=326) | 3.33±0.82 | 16 (4.9) | 26 (8.0) | 118 (36.2) | 166 (50.9) |
| 6 | I know how to read and write in my native language (n=326) | 3.71±0.72 | 13 (4.0) | 14 (4.3) | 27 (8.3) | 272 (83.4) |
| 7 | I attend social functions with people from my native country (n=326) | 3.17±0.96 | 31 (9.5) | 36 (11.0) | 104 (31.9) | 155 (47.5) |
| 8 | I speak my native language at home (n=326) | 3.76±0.61 | 7 (2.1) | 10 (3.1) | 37 (11.3) | 272 (83.4) |
| 9 | I regularly read magazines of my ethnic group (n=326) | 2.13±1.14 | 140 (42.9) | 56 (17.2) | 77 (23.6) | 53 (16.3) |
| 10 | I know how to speak my native language (n=326) | 3.80±0.55 | 4 (1.2) | 11 (3.4) | 32 (9.8) | 279 (85.6) |
| 11 | I am familiar with the history of my native country (n=326) | 3.44±0.84 | 19 (5.8) | 18 (5.5) | 88 (27.0) | 210 (61.7) |
| 12 | I like to listen to music of my ethnic group (n=326) | 2.88±1.16 | 66 (20.2) | 45 (13.8) | 77 (23.6) | 138 (42.3) |
| 13 | I like to speak my native language (n=326) | 3.65±0.74 | 13 (4.0) | 13 (4.0) | 50 (15.3) | 250 (76.7) |
| 14 | I speak my native language with my spouse or partner (n=326) | 2.92±1.30 | 89 (27.3) | 22 (6.7) | 41 (12.6) | 174 (53.4) |
| 15 | When I pray, I use my native language (n=326) | 3.07±1.22 | 72 (22.1) | 17 (5.2) | 54 (16.6) | 183 (56.1) |
| 16 | I stay in close contact with my family members and relatives in my native country (n=326) | 3.45±0.90 | 23 (7.1) | 23 (7.1) | 64 (19.6) | 216 (66.3) |
| 17 | I am informed about current affairs in Hungary (n=326) | 2.63±0.89 | 48 (14.7) | 70 (21.5) | 164 (50.3) | 44 (13.5) |
| 18 | I feel totally confident with Hungarian people (n=326) | 2.50±0.98 | 61 (18.7) | 97 (29.8) | 113 (34.7) | 55 (16.9) |
| 19 | I have many Hungarian acquaintances (n=326) | 2.02±0.97 | 124 (38.0) | 102 (31.3) | 71 (21.8) | 29 (8.9) |
| 20 | I feel home in Hungary (n=326) | 2.69±0.95 | 50 (15.3) | 66 (20.2) | 146 (44.8) | 64 (19.6) |
| 21 | I feel accepted by Hungarians (n=326) | 2.62±0.98 | 51 (15.6) | 91 (27.9) | 115 (35.3) | 69 (21.2) |
| 22 | I know how to prepare Hungarian foods (n=326) | 1.90±1.02 | 157 (48.2) | 73 (22.4) | 66 (20.2) | 30 (9.2) |
| 23 | I regularly read a Hungarian newspaper (n=326) | 1.52±0.88 | 228 (69.9) | 44 (13.5) | 38 (11.7) | 16 (4.9) |
| 24 | I speak Hungarian at home (n=326) | 1.45±0.89 | 247 (75.8) | 32 (9.8) | 26 (8.0) | 21 (6.4) |
| 25 | I am familiar with important people in Hungarian history (n=326) | 2.00±0.99 | 131 (40.2) | 91 (27.9) | 76 (23.3) | 28 (8.6) |
| 26 | I think in Hungarian (n=326) | 1.36±0.81 | 263 (80.7) | 24 (7.4) | 24 (7.4) | 15 (4.6) |
| 27 | I speak Hungarian with my spouse or partner (n=326) | 1.34±0.81 | 271 (83.1) | 17 (5.2) | 21 (6.4) | 17 (5.2) |
| 28 | I like to eat Hungarian foods (n=326) | 2.59±1.09 | 73 (22.4) | 69 (21.2) | 102 (31.3) | 82 (25.2) |

Items number 1 to 16 are the questions about ethnic society immersion (ESI)

Items number 17 to 28 are the questions about dominant society immersion (DSI)

Additional file 2

**Alpha Cronbach’s ESI**

| **Case Processing Summary** | | | |
| --- | --- | --- | --- |
|  | | N | % |
| Cases | Valid | 15 | 100.0 |
|  | Excluded^a^ | 0 | .0 |
|  | Total | 15 | 100.0 |
| a. Listwise deletion based on all variables in the procedure. | | | |

| **Reliability Statistics** | |
| --- | --- |
| Cronbach's Alpha | N of Items |
| .819 | 16 |

| **Item-Total Statistics** | | | | |
| --- | --- | --- | --- | --- |
|  | Scale Mean if Item Deleted | Scale Variance if Item Deleted | Corrected Item-Total Correlation | Cronbach's Alpha if Item Deleted |
| 1. I speak my native language with my friends and acquaintances from my country of origin. | 49.73 | 45.638 | .509 | .808 |
| 2. I have never learned to speak the language of my native country. | 51.87 | 46.124 | .195 | .825 |
| 3. I eat traditional food from my native culture. | 50.07 | 44.781 | .256 | .823 |
| 4. I feel comfortable speaking my native language. | 49.40 | 47.829 | .416 | .816 |
| 5. I am informed about current affairs in my native country. | 49.67 | 47.952 | .176 | .821 |
| 6. I know how to read and write in my native language. | 49.60 | 45.686 | .283 | .818 |
| 7. I attend social functions with people from my native country. | 49.60 | 44.829 | .528 | .806 |
| 8. I speak my native language at home. | 49.53 | 43.838 | .482 | .806 |
| 9. I regularly read magazines of my ethnic group. | 51.47 | 39.838 | .583 | .797 |
| 10. I know how to speak my native language. | 49.33 | 49.381 | .000 | .823 |
| 11. I am familiar with the history of my native country. | 49.87 | 43.124 | .508 | .804 |
| 12. I like to listen to music of my ethnic group. | 49.87 | 39.552 | .653 | .792 |
| 13. I like to speak my native language. | 49.40 | 47.829 | .416 | .816 |
| 14. I speak my native language with my spouse or partner. | 50.80 | 33.886 | .755 | .781 |
| 15. When I pray, I use my native language. | 50.13 | 38.124 | .618 | .794 |
| 16. I stay in close contact with my family members and relatives in my native country. | 49.67 | 44.381 | .562 | .804 |

**Alpha Cronbach’s DSI**

| **Case Processing Summary** | | | |
| --- | --- | --- | --- |
|  | | N | % |
| Cases | Valid | 15 | 100.0 |
|  | Excluded^a^ | 0 | .0 |
|  | Total | 15 | 100.0 |
| a. Listwise deletion based on all variables in the procedure. | | | |

Additional file 3

| **Reliability Statistics** | |
| --- | --- |
| Cronbach's Alpha | N of Items |
| .880 | 12 |

| **Item-Total Statistics** | | | | |
| --- | --- | --- | --- | --- |
|  | Scale Mean if Item Deleted | Scale Variance if Item Deleted | Corrected Item-Total Correlation | Cronbach's Alpha if Item Deleted |
| 1. I am informed about current affairs in Hungary. | 20.67 | 54.095 | .309 | .887 |
| 2. I feel totally confident with Hungarian people. | 20.60 | 50.400 | .534 | .873 |
| 3. I have many Hungarian acquaintances. | 21.33 | 47.381 | .777 | .857 |
| 4. I feel home in Hungary. | 20.73 | 48.781 | .555 | .873 |
| 5. I feel accepted by Hungarians. | 20.47 | 49.695 | .567 | .871 |
| 6. I know how to prepare Hungarian foods. | 21.93 | 50.352 | .759 | .862 |
| 7. I regularly read a Hungarian newspaper. | 22.00 | 51.429 | .598 | .869 |
| 8. I speak Hungarian at home. | 22.13 | 51.695 | .687 | .866 |
| 9. I am familiar with important people in Hungarian history. | 21.53 | 47.124 | .732 | .860 |
| 10. I think in Hungarian. | 22.33 | 59.952 | .000 | .887 |
| 11. I speak Hungarian with my spouse or partner. | 22.00 | 51.714 | .574 | .871 |
| 12. I like to eat Hungarian foods. | 20.93 | 48.495 | .703 | .862 |

**Regression- multicollinearity**

| **Model Summary** | | | | |
| --- | --- | --- | --- | --- |
| Model | R | R Square | Adjusted R Square | Std. Error of the Estimate |
| 1 | .328^a^ | .107 | .070 | .620 |
| a. Predictors: (Constant), 42. visit psychologist after, alcohol after, 9. Ethnic minority, year of study dik, general health coba, Religion 4 categories, marital Status dik, 3. Gender, economic status dik, smoking after, PSS dik, 2. Age, mental health coba | | | | |

| **ANOVA^a^** | | | | | | |
| --- | --- | --- | --- | --- | --- | --- |
| Model | | Sum of Squares | df | Mean Square | F | Sig. |
| 1 | Regression | 14.411 | 13 | 1.109 | 2.884 | .001^b^ |
|  | Residual | 119.947 | 312 | .384 |  |  |
|  | Total | 134.359 | 325 |  |  |  |
| a. Dependent Variable: ***Acculturation Categories (***Integration vs Separation***)*** | | | | | | |
| b. Predictors: (Constant), 42. visit psychologist after, alcohol after, 9. Ethnic minority, year of study dik, general health coba, Religion 4 categories, marital Status dik, 3. Gender, economic status dik, smoking after, PSS dik, 2. Age, mental health coba | | | | | | |

| **Coefficients^a^** | | | | | | | | |
| --- | --- | --- | --- | --- | --- | --- | --- | --- |
| Model | | Unstandardized Coefficients | | Standardized Coefficients | t | Sig. | Collinearity Statistics | |
|  |  | B | Std. Error | Beta |  |  | Tolerance | VIF |
| 1 | (Constant) | 1.735 | .317 |  | 5.481 | .000 |  |  |
|  | 2. Age | -.019 | .013 | -.083 | -1.430 | .154 | .847 | 1.180 |
|  | 3. Gender | .169 | .070 | .132 | 2.409 | .017 | .958 | 1.044 |
|  | year of study dik | .246 | .080 | .178 | 3.077 | .002 | .858 | 1.166 |
|  | economic status dik | -.012 | .081 | -.008 | -.143 | .886 | .931 | 1.074 |
|  | 9. Ethnic minority | .065 | .102 | .036 | .638 | .524 | .908 | 1.101 |
|  | marital Status dik | -.160 | .082 | -.108 | -1.959 | .051 | .941 | 1.062 |
|  | Religion 4 categories | .094 | .030 | .170 | 3.139 | .002 | .971 | 1.030 |
|  | smoking after | .016 | .064 | .014 | .256 | .798 | .908 | 1.101 |
|  | alcohol after | -.058 | .069 | -.048 | -.847 | .397 | .905 | 1.105 |
|  | mental health coba | -.031 | .110 | -.018 | -.280 | .780 | .680 | 1.470 |
|  | general health coba | .317 | .186 | .097 | 1.700 | .090 | .888 | 1.127 |
|  | PSS dik | .106 | .104 | .063 | 1.023 | .307 | .757 | 1.320 |
|  | 42. visit psychologist after | -.107 | .104 | -.058 | -1.031 | .303 | .905 | 1.105 |
| a. Dependent Variable: kategori mean dari data | | | | | | | | |
